# Anti-inflammatory effects of zinc in PMA-treated human gingival fibroblast cells

**DOI:** 10.4317/medoral.19896

**Published:** 2015-02-07

**Authors:** Jisun Kim, Sangwoo Kim, Sangmi Jeon, Zheng Hui, Young Kim, Yeonggwan Im, Wonbong Lim, Changsu Kim, Hongran Choi, Okjoon Kim

**Affiliations:** 1 Department of Oral Pathology, Dental Science Research Institute and Medical Research Center for Biomineralization Disorders, School of Dentistry, Chonnam National University, Bug-Gu, Gwangju, Korea; 2 Department of Oral Pathology, Chonnam National University hospital, Bug-Gu, Gwangju, Korea; 3 Department of oral medicine, School of Dentistry, Chonnam National University, Gwangju, Republic of Korea; 4 Su Dental Clinical Research Center, Yeoksam-dong, Gangnam-gu, Seoul, Korea

## Abstract

Objectives: Abnormal cellular immune response has been considered to be responsible for oral lesions in recurrent aphthous stomatitis. Zinc has been known to be an essential nutrient metal that is necessary for a broad range of biological activities including antioxidant, immune mediator, and anti-inflammatory drugs in oral mucosal disease. The objective of this study was to investigate the effects of zinc in a phorbol-12-myristate-13-acetate (PMA)-treated inflammatory model on human gingival fibroblast cells (hGFs). Study Design: Cells were pre-treated with zinc chloride, followed by PMA in hGFs. The effects were assessed on cell viability, cyclooxygenease-1,2(COX-1/2) protein expression, PGE2 release, ROS production and cytokine release, Results: The effects were assessed on cell viability, COX1/2 protein expression, PGE2 release, ROS production, cytokine release. The results showed that, in the presence of PMA, zinc treatment leads to reduce the production of ROS, which results in decrease of COX-2 expression and PGE2 release. Conclusions: Thus, we suggest that zinc treatment leads to the mitigation of oral inflammation and may prove to be an alternative treatment for recurrent aphthous stomatitis.

** Key words:**Zinc, inflammatory response, cytokines, phorbol-12-myristate-13-acetate, gingival fibroblasts cells.

## Introduction

Recurrent aphthous stomatitis (RAS) is a common oral mucosal disorder characterized by recurrent painful oral aphthaes. The prevalence of RAS is between 10%~20% in the general population (1,2). RAS is an inflammatory condition of unknown etiology. Various cytokines such as interleukin-6 (IL-6) and IL-8 have been implicated as potential etiopathogenic agents (3-5). RAS progression is regulated by the host response, and there is increasing evidence that non-immune cells such as human gingival fibroblasts (hGFs), participate in the host response (6). Other studies have reported that hGFs express Toll-like receptors and CD14, which is consistent with the finding that these cells respond to lipopolysaccharide (7). Arikan *et al*. demonstrated that lipid per oxidation seems to play a crucial role in the pathogenesis of recurrent aphthous stomatitis (8). RAU has known to evoke the Immune alterations by the activation of T lymphocytes and cytokines secretion (9). There have also been reported the dysfunction of enzyme super oxide dismutase (SOD), which participates in the inflammatory response of RAS (10). Recent studies reported that phorbol 12-myristate 13-acetate (PMA) could play an important role in the induction of cyclooxygenease-2(COX-2) expression and cytokines release (11-13). The synthesis of prostaglandin (PGs) in particular prostaglandin E2(PGE2) is regulated by main enzyme isoforms, cyclooxygenease-1(COX-1) and COX-2 (14). The PMA has known to induce the reactive oxygen species (ROS) mediated apoptosis in chronic inflammatory disease (15). It is well known that oxidative stress can arise through the increased production of reactive oxygen species (ROS) and/or a deficiency of antioxidant defenses. Insufficient antioxidant enzyme synthesis may in turn be due to decreased micro nutrient availability (8). Hence dietary intake of antioxidants is indispensable to protecting against oxidative-derived diseases. Increasing attention has been focused on the role of ROS produced by activated neutrophils during the inflammatory response (16).

To improve the deficient antioxidant synthesis, several clinical studies have suggested that zinc treatment could be useful to balance the redox potential (17,18). Zinc, thus not only function as an antioxidant but is also an anti-inflammatory agent. Zinc ions regulated T- and B-lymphocyte function, making it vital for the maintenance of normal immune function and resistance to infection (19,20). Zinc supplementation has been shown to have a favorable effect on fibrosis (21,22), metabolism in the thyroid gland (23), anti-oxidation(18), and anti-depression (24). Zinc is an essential nutrient that is necessary for a broad range of biological activities. To date, more than 300 zinc-containing enzymes have been identified. Zinc deficiency causes biological dysfunction of gene expression, protein synthesis, immunity, skeletal growth and maturation, gonadal development, pregnancy, taste perception, and appetite (9,25-29). Some researchers have investigated the possible correlation of some oxidative stress parameters in Behcet’s disease and reported that zinc levels were inversely correlated with the clinical manifestation index (30,31), which demonstrated the importance of zinc as an antioxidant in inflammation-induced cells. Increasing evidence demonstrates that zinc has an anti-inflammatory effect (32). An earlier report showed that injection with zinc sulfate reduced LPS-induced teratogenicity in mice (19) and a recent study found that injection with zinc sulfate alleviated LPS-induced neurodevelopmental damage in the fetal brain (33). Although previous studies have reported that zinc can reduce inflammation, it is unclear which cytokines are involved and reduced by zinc in the inflammatory state.

The purpose of the present study was to investigate the effect of zinc on the process of wound healing in human gingival fibroblasts cells on an in vitro Phorbol-12-myristate-13-acetate (PMA)-induced inflammatory model. Also, the activation profiles of inflammatory cytokines were revealed in response to zinc treatment.

## Material and Methods

- Chemicals and Reagents

Phorbol-12-myristate-13-acetate (PMA), Zinc chloride (ZnCl2), 3-(4,5-dimethylthiazolyl-2)-2,5-diphenyltetrazolium bromide (MTT), dimethyl sulfoxide (DMSO) and 2’, 7’-dichlorodihydrofluorescein diacetate (H2DCF-DA), Trisma base, sodium azide, sodium chloride and phenylmethylsulphonyl fluoride were purchased from Sigma (St. Louis, Mo, USA). Protein inhibitor cocktail were purchased from roche (roche, Mannheim, Germany).

- Primary Cell Culture 

Human gingival fibroblast cells (hGFs) were obtained from three healthy adults visiting the Chonnam National University Hospital for a gingivectomy. The gingival tissues were finely cut with scissors and cultured in an alpha minimum essential medium (α-MEM) (GibcoBRL, Rockville, MD, USA) supplemented with 10% heat-inactivated fetal bovine serum and 1% antibiotic–antimycotic solution (Cambrex Bio Science, Baltimore, MD, USA) at 37 °C in a 5% CO2 humidified chamber. The medium was replaced with fresh medium and the adherent hGFs were allowed to reach approximately 70% confluence. The cells were then detached using trypsine-ethylenediamine tetra acetic acid (trypsin-re TA: GibcoBRL, Rockville, MD, USA) solution and plated again (subcultured) in 6-well plates for each experiment.

- Cell viability assay

The cell viability was evaluated using the (3-(4,5-dimethylthiazol-2-yl)-2,5-diphenyltetrazolium bromide) (MTT; Sigma–Aldrich) assay. The cells were seeded at 5 x 103 cells per 200 uL of medium in 96-well plates, and cultured for 1 day at 37 °. Various concentrations (0,1,5,10,20, and 50 uM) of ZnCl2 for a zinc donor were added in each well with phorbol 12-myristate 13-acetate (PMA, 1uM), and the cells were subsequently incubated at 37° for 24 hours. After removing the medium, 100 uL of MTT (50 ug/ml) was added to the cells. The cells were incubated at 37° for 4 hours and 30 minutes to allow color development, and the formazan product was solubilized by the addition of 50 ml dimethyl sulfoxide (Calbiochem Bio-Mol, La Jolla, CA). Optical density was measured at 570 nm and reference with 655nm using a microplate reader (Bio-Rad, Hercules, CA).

- Western blotting

The hGFs were seeded at 5 x 104 cells per well in ά-MEM media, and cultured for 1 day. The ZnCl2 (1,5,10 and 20 uM) was then added to each well in the presence of PMA (1uM), and the cells were incubated for 24 hours. The medium was removed and washed twice with PBS. Cell lysates were then prepared in 200 ul of cold lysis buffer (1% NP-40,50 mM Tris–HCl, pH 7.5, 150 mM NaCl, 0.02% sodium azide, 150 mg/ml phenylmethylsulphonyl fluoride, protein inhibitor cocktail). Thirty milligrams of cell lysates were separated in a 10% sodium dodecyl sulfate polyacrylamide gel and transferred onto a polyvinylidene difluoride membrane (Amersham, Arlington Heights, IL). The membrane was blocked with blocking solution [5% skim milk in Tris-Buffered Saline and Tween 20(TBST) (2.42 g/L Tris–HCl, 8 g/L NaCl, 0.1% Tween 20, pH 7.6)] for 30 minutes and rinsed briefly in TBST. The membrane was incubated overnight at 4° with anti-COX-1 (1:1000; Santa Cruz Biotechnologies, Santa Cruz, CA), anti-COX-2 (1:1,000; Abcam, Cambridge, UK), and Glyceraldehyde 3-phosphate dehydrogenase (GAPDH) antibody (1:2,500; Santa Cruz). After rinsing with TBST, the membrane was incubated for 1 hour with anti-rabbit and anti-mouse horse radish peroxidase-conjugated (1:2,000) secondary antibody. Finally, the membrane was washed in TBST, and the immunoreactivities of the proteins were detected using an enhanced chemiluminescence detection kit (Amersham) and the levels were determined by densitometric analysis using Scion Image software (Scion Corp, Frederick, MD).

- Enzyme-Linked Immunoassay for PGE2

The PGE2 expression level, the main metabolite of COX, was used in all subsequent experiments for this study. The amount of PGE2 was measured in the supernatants using a commercially available enzyme immunoassay kit (R&D System, Minneapolis, MN, USA) according to the manufacturer’s instruction. The hGFs were seeded at 5 x 104 cells per well in ά-MEM media, and cultured for 1 day. The ZnCl2 (1, 5, 10 and 20 uM) was then treated to each well in the presence of PMA (1uM), and the cells were incubated for 24 hours. After 24 hr incubation, the absorbance for PGE2 was measured at 586 nm by using a colorimetric micro plate reader (Bio-rad, Hercules, CA, USA).

- Detection of total ROS formation with flow cytometry 

Total reactive oxygen species (ROS) were assayed using 2’, 7’-dichlorodihydrofluorescein diacetate (H2DCF-DA). DCF-DA enters cells passively, where it is enzymatic ally deacetylated by esterases to the non-fluorescent 2, 7- dihydrodichlorofluorescein (DCF-H). In the presence of oxidizing molecules such as O2-, DCF-H is converted to the highly fluorescent DCF. To measure intracellular ROS levels, the hGFs were seeded at 5 x 104 cells per well in media, and cultured for 1 day. The ZnCl2 (1,5,10 and 20 uM) was then treated to each well in the presence of PMA (1uM), and the cells were incubated for 24 hours. After incubation, the cells were washed with PBS containing 10 nM glucose and treated with 10 mM H2DCF-DA for 20 minutes. The cells were detached using trypsin-EDTA solution, and ROS levels were analyzed using flow cytometry (Beckman Coulter Fullerton, CA) at 485 nm excitation and with 530 nm emission filters. To monitor the ROS formation, cells grown on cover slips were incubated with 10 nM of DCF-DA for 20 minutes. After the cells were washed with PBS containing 10 mM glucose, DCF fluorescence intensity was monitored using a confocal microscope (Carl Zeiss, Oberkochen, Germany) set at excitation and emission wavelengths of 488 and 525 nm, respectively.

- Cytokine profiling

To assess cytokine production profiles, the supernatant from the cultured hGFs was collected and assayed using a human inflammation antibody array (R&D Systems, Minneapolis, MN, U.S.A.) according to the instruction manual. This method uses a membrane coated with specific antibodies for each cytokine forming an array. The cytokine signal was detected using an ECL detection kit (Promega, Medison, WI, USA) and quantified by densitometric analysis using Scion Image software (Scion Corp, Frederick, MD, USA). The signal intensity of each spot was adjusted to the corresponding internal control provided for each membrane by the manufacturer (cytokine profiling). The ratio of expression was calculated by dividing the signal intensity of each cytokine by the signal intensity of the internal control for each sample.

- Statistics

Data is expressed as the mean ± standard deviation. All experiments were carried out three times. The differences between the groups were evaluated using one way ANOVA. Data were considered statistically significant, at *P* < 0.05.

## Results

- The cell survival effects of zinc in PMA-treated hGFs

**Figure 1:**
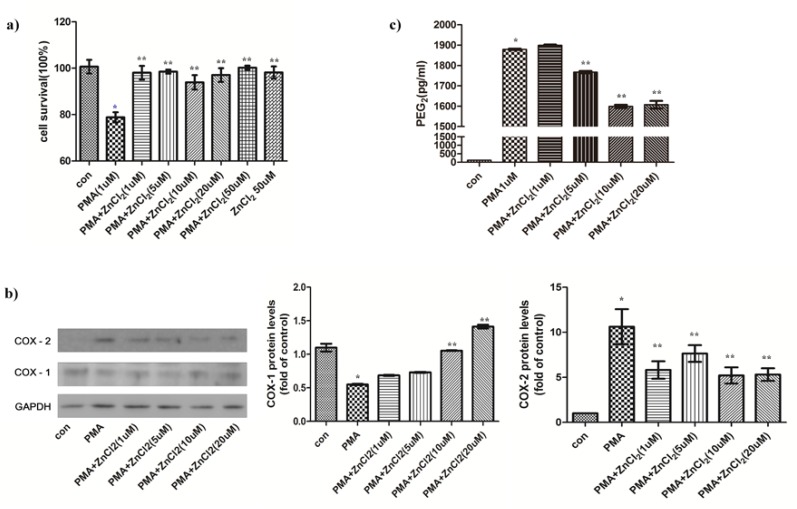
Effects of zinc on PMA-treated cell survival, COX-1/2 protein expressions and PGE2 release in hGFs. (a) The cell viability was assessed using an MTT assay for the indicated concentrations of ZnCl2 (0, 1, 5, 10, 20, and 50 µM) in the presence of PMA (1 µM). Data are reported as the mean ± SD (n = 3). (b) The cyclooxygenase -1/2 (COX-1/2) expressions and COX-2 expression level in hGFs were represented. The cells in each group were prepared for immunodetection with anticyclooxygenase-1 or 2 antibodies. The membranes were denuded and GAPDH was employed to load the same protein concentration. Densitometric analysis of COX-2 represents the mean ratio ± SD from three separate experiments. Significant differences are indicated as *p < 0.05 compared to PMA. (c) Released PGE2 was measured from recuperation of the culture medium from the previously mentioned condition, and these were processed for analysis by ELISA.

To determine the effects of Zinc on cell survival in PMA-treated hGFs, cells were treated by 1μM of PMA with or without Zinc in a dose dependent manner. In the PMA-treated hGFs, cell survival was decreased to 80% as shown in fig. 1a. However, zinc could recover the cell survival to around 100% of control.

- The effects of zinc on COX protein expression and PGE2 release in PMA-treated hGFs

The effect of zinc was investigated on COX protein expression in PMA-induced hGFs using western blot analysis. Protein expression of COX-2 after PMA treatments was revealed in the hGFs (Fig. 1b). After the zinc treatment, COX-2 expression was significantly decreased to against PMA induced inflammatory response (Fig. 1b). However, PMA induced decrease of COX-1 expression, while ZnCl2 supplementation recovered to control levels (Fig. 1b). The release of PGE2 was increased to 1850 pg/mL in PMA-treated hGFs (Fig. 1c). On the other hand, PGE2 release was reduced to 1600 pg/mL in the presence of zinc (20 μM).

- The effects of zinc on ROS levels in PMA-treated hGFs

**Figure 2:**
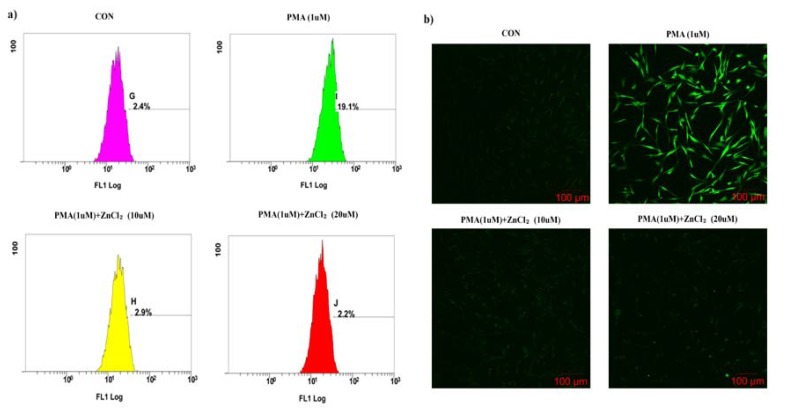
Effect of zinc on PMA - treated ROS generation in hGFs. (a) The DCF fluorescence distribution is represented by flow cytometry histograms (X axis: log of the fluorescence intensity; Y-axis: cell number). (b) Green fluorescence of DCF-DA, indicating intracellular ROS formation, was detected by confocal microscopy. All magnifications are × 200.

To assess the effects of zinc on intracellular ROS levels in the PMA-treated hGFs, the production of intercellular ROS levels were measured using DCF-DA fluorescence. As shown in fig. 2a, DCF-DA fluorescence was increased by PMA to 83.7% in hGFs. However, zinc (20μM) on PMA-treated cells decreased the DCF-DA fluorescence to 67.5%. To confirm this result, we compared to between PAM treatments only and ZnCl2 supplemented groups by confocal microscopy (Fig. 2b). Significant DCF fluorescence was seen in the PMA-treated hGFs, but Zinc supplemented groups did not have DCF fluourescence in the zinc (10μM & 20μM) treated with PMA.

- The effects of zinc on cytokine production in PMA-treated hGFs 

**Figure 3:**
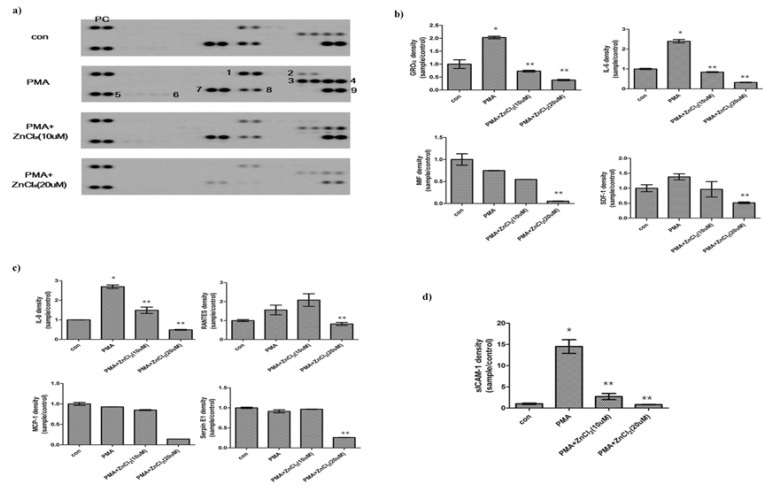
Effect of zinc on PMA - treated cytokines release in hGFs. (a) Expression of cytokine release in the presence or absence of zinc on PMA-induced hGFs. (PC, Positive Control; 1, GRO-alpha; 2, sICAM-1; 3, IL-6; 4, IL-8; 5, RANTES; 6, SDF-1; 7, MCP-1; 8, MIF, 9, SerpinE1). (b) Innate cytokines, (c) chemokines, and (d) not-distinguished cytokines were represented. For investigation of released cytokines, supernatants was harvested after 24 hr and then assayed using a cytokine profile array kit. Bars represent the mean ratio ± SD from the 2 experiments. Significant differences are indicated as *p< 0.05 compared to PMA.

To assess cytokine production, supernatants for hGFs cultured with zinc in the presence of PMA were collected after 24 hr. Cytokine profiles were assessed using a cytokine antibody array that can detect 40 different basal levels of a number of cytokines (Fig. 3a.). In the results, four innate cytokines, four chemokines, and one not-distinguished (N.D.) cytokine were represented.

Among these cytokines, growth-regulated oncogene-alpha (GROα), interleukin-6 (IL-6), macrophage migration inhibitory factor (MIF), and stromal cell-derived factor-1(SDF-1) of innate cytokines were measured in the media (Fig. 3b). GROα and SDF1 levels were increase by PMA, however, zinc supplements decrease to control levels or lower of control. IL-6 showed the greatest amount of release in response to PMA, increasing significantly to 2.4-fold of the control level. However, zinc (20 μM) led to a decrease in IL-6 release; reducing the level to 0.5-fold of the control. MIF levels were decreased by PMA. In zinc supplements with PMA, MIF levels were decrease more comparing to PMA treatments only.

Of the chemokine, interleukin-8 (IL-8), regulated on activated normal T-cell expressed and secreted (RANTES), monocyte chemoattractant protein-1 (MCP-1), and serpin E1 were assayed (Fig. 3c). IL-8, an important mediator of the innate immune response, was increased significantly to 2.7-fold the level of controls in PMA treated hGFs. In the PMA + zinc (20uM) group, IL-8 was significantly decreased to the half level of controls. MCP-1 and Serpine E1 were diminished to about 0.2-fold of the unchallenged controls after zinc (20 uM) treatment in the presence of PMA. PMA induced RANTES levels to 1.5-fold of control. At 20uM, zinc attenuated RANTES levels which were induced by PMA.

In N.D., sCIAM-1 was significantly increased to 15-fold the level of control in PMA-treated-hGFs (Fig. 3d). However, zinc led to a decrease in sICAM-1 release, reducing the levels to those of the control cells.

## Discussion

Although principle etiology of oral inflammatory disease such as RAS is unclear, some researchers have proposed aphthous formation as a cause due to oxidative stress. (8,34). Recurrent aphthous stomatitis (RAS) is characterized by the appearance of initially necrotic ulcers (9). Immune alterations have been observed resulting in the activation of T lymphocytes, cytokines secretion (9). Changes have also been reported in elements of the salivary defense system, such as the enzyme superoxide dismutase (SOD), which participates in the inflammatory response of RAS (10). In inflammatory model, PMA has the function of cell death and inflammatory response by cytokines regulation such as IL-6, IL-8 and T-cell activation including our results (12,35) . We suggested that PMA initiated inflammatory response to contribute to its pathogenesis.

Zinc therapy has been used extensively in clinical dentistry. Moreover, zinc is an important component of restorative materials and is also used as an active component in toothpaste and mouth-rinses. Clinical studies have shown that zinc is several beneficial effects such as antioxidant and anti-inflammatory properties in psoriasis, hair loss, wound healing, leg ulcers (36-38). Moreover, zinc is an essential element and a well-established antioxidant and zinc deficiency has been reported to be a potential risk factor for oral disease (39), (40). Chen and Liao *et al*. suggest that zinc can have either positive or negative effects on the physiology of cells depending on the local concentration, localization, and/or state (40,41). In this study, zinc ions supplementation could be apply oral disease by inflammatory response such as aphthous ulcer, aphthous stomatitis.

In the present study, hGFs were treated with PMA to induce inflammation for an in-vitro experiment model. A inducible COX-2 expression in quiescent fibroblasts in response to stimulation with PMA is known to signal via protein kinase C (PKC) and its downstream signaling molecule, mitogen activated kinase (MAPK) (42,43). The treatment of PMA (1uM) subsequently led to the ROS generation, COX-2 expression, PGE2 release, and cell viability decrease. The ability of zinc to reduce inflammation is known that zinc could lead the intracellular ROS scavenging and relive cellular oxidative stress. In present study, zinc treatment was shown to scavenge intracellular ROS, which mediate COX-2 expression, and inhibit the release of PGE2. COX protein played different roles according to the cause of inflammatory cause of inflammatory response. COX-2 appears to be the dominant source of prostaglandin formation in inflammation. Increase of COX-2 expressed on provocation of inflammatory cells. COX-2 regulation usually treated to chronic inflammatory diseases (44). COX-1 is constitutively expressed in resident inflammatory cells, and there is evidence for induction of COX-1 during LPS-mediated inflammatory response and cellular differentiation (44). However, the function of COX protein is not understood yet.

Given the antioxidant capacity of zinc on oxidative stress, zinc might protect cells indirectly against ROS and subsequently relieve the anti-inflammatory reaction involving the COX-2 and PGE2 pathway. Other reports confirm that serum zinc levels in patients with RAS were significantly lower than in controls (45). Low zinc levels in our patients may be probably due to the increased consumption of zinc: (1) the continuous process of ulcer healing; (2) because of the antioxidant activity of zinc as it may act as free radical scavenger; (3) existence of completion in intestinal absorption; and the inverse relationship between copper and zinc concentration (46). We suggested that aphthous ulcers were chronic inflammatory disease by COX-2 and PGE2. Moreover, Zinc supplementation might apply treatments of inflammatory response by ROS scavenge in zinc deficiency patients.

Cytokines and chemokines are known to play a pivotal role in the immunopathology of a number of immune diseases, and release of appropriate cytokine is essential for the initiation and effector stages of immunity and inflammation (47). The level of inflammatory cytokines could reflect process and development of oral disease in patient (48). Zinc treatment significantly decreased IL-6 and IL-8 release in PMA-treated hGFs. Their function is mediated by primary cytokines such as IL-1beta and TNF-alpha, which is secreted within minutes in response to stimuli (49). These kinds of cytokines lead to increased vascular permeability to IL-6 and recruit the leukocytes to the region, including those expressing IL-8 (50). The fact that IL-6 and IL-8 were increased in PMA-treated hGFs indicates that primary cytokines were effective in immediately enhancing the inflammatory response, so that the IL-6 and IL-8 levels are elevated in an autocrine manner. On the other hand, the IL-6 and IL -8 levels were decreased by zinc, resulting from autocrine suppression of the inflammatory response. IL-6 and IL-8 

In this study, MCP-1 and Serpine E1 were significantly decreased by zinc treatment in the presence of PMA. Also, RANTES release was recovered from a higher level in PMA-treated hGFs. RANTES, MCP-1, and Serpin E1 are chemokines produced in response to stimulation by cytokines such as IL-6 and TNF-α, and are secreted by a variety of cells, including vascular smooth muscle cells (VSMCs), epithelial cells, T cells, macrophages, and platelets (51,52). RANTES and MCP- 1 belong to a chemotactic cytokine family and are responsible for the chemotactic migration and activation of neutrophils and other cell types (such as monocytes, lymphocytes, basophils, and eosinophils) at sites of inflammation. The decrease of RANTES, MCP-1, and Serpine E1 by zinc treatment in presence of PMA is thus reasonable to suggest that zinc probably contribute to relieve the recruitment of inflammatory cells in the periodontal tissue. Previously, it has been reported that gingival fibroblasts produce the alpha chemokine, IL-8, upon stimulation by lipopolysaccharide or IL-1β. These findings would suggest that by producing RANTES/CCL5 and IL-8, human gingival fibroblasts might participate in the regulation of the local inflammatory process during both the acute and the chronic stages of inflammation during RAS (53,54).

Considering the anti-inflammatory effects of zinc based on the present study, zinc reduces the ROS generation and decreases COX-2 expression and PGE2 release in PMA-treated hGFs. Based on these result, the inflammatory cytokine profile after zinc treatment indicates that zinc leads the decrease of the release in secondary pro-inflammatory cytokines and chemotactic cytokine family for the recruitment of inflammatory cells in the periodontal tissue. This evidence suggests that zinc mitigates inflammation and may be clinically useful as an anti-inflammatory treatment on RAS.
